# Prevalence of Breastfeeding: Findings from the First Health Service Household Interview in Hunan Province, China

**DOI:** 10.3390/ijerph14020150

**Published:** 2017-02-04

**Authors:** Hong Qin, Lin Zhang, Lingling Zhang, Wei Zhang, Li Li, Xin Deng, Danping Tian, Jing Deng, Guoqing Hu

**Affiliations:** 1 Xiangya School of Public Health, Central South University, 110 Xiangya Road, Changsha 410078, Hunan Province, China; qinhong@csu.edu.cn (H.Q.); zhanglinxysm@163.com (L.Z.); zhangweizw@gmail.com (W.Z.); cslilee1009@gmail.com (L.L.); dengxin66@126.com (X.D.); danpingtian@foxmail.com (D.T.); dengjing2@126.com (J.D.); 2 Department of Public Health Sciences, Clemson University, Clemson, SC 29634, USA; linglz@clemson.edu

**Keywords:** breastfeeding, exclusive breastfeeding, prevalence, China

## Abstract

*Background:* With the development of economy and urbanization, methods of child-feeding have significantly changed in China over the past three decades. However, little is known about breastfeeding in China since 2009. This study aims to update information on the prevalence of breastfeeding in China. *Methods:* Data were obtained from the first Health Service Household Interview Survey of Hunan Province, China. Of 24,282 respondents, 1659 were aged five years or younger. We ran multivariable logistic regression to examine the impact of urban/rural setting, gender, age and household income per capita on the use of breastfeeding. *Results:* A total of 79.4% of children aged 5 years or younger had been breastfed at some point and 44.9% been breastfed exclusively in the first 6 months of life. After controlling for setting urban/rural setting, gender and child age, children from households with average family income were more likely to be breastfed than those from households with the lowest family income (adjusted odds ratio: 2.28). Children from households with higher and the highest family income were less likely to be exclusively breastfed in the first 6 months of life compared to those from households with the lowest family income (adjusted odds ratio: 0.51 and 0.68, respectively). *Conclusions:* It is encouraging that the prevalence of exclusive breastfeeding for infants in the first 6 months of life in Hunan Province, China is approaching the goal of 50% proposed by the World Health Organization (WHO). Nevertheless, more efforts are needed to further promote exclusive breastfeeding in the first 6 months after birth.

## 1. Introduction

Breastfeeding can provide better health outcomes by preventing diseases and promoting health in the short and long term for both mothers and children [1,2,3,4,5]. It is estimated that approximately 800,000 children’s lives could be saved globally each year if every child was exclusively breastfed in their initial 6 months of life [6]. Although the World Health Organization (WHO) recommends exclusive breastfeeding, roughly 43% of infants aged 0–6 months old were exclusively breastfed globally in 2015, which indicated a significant gap in terms of infant health [7]. 

Exclusive breastfeeding was the predominant method of child feeding in China 50 years ago; at that time half of infants were exclusively breastfed during the first 12 months of life [8]. With the economy boom and urbanization, however, methods of child feeding have significantly changed in China over the past three decades. Previous studies have shown a much lower prevalence of exclusive breastfeeding in infants—35% of infants under 4 months of age were exclusively breastfed in Jinan City, China (2000) [8]; less than 5% infants were exclusively breastfed in the first 6 months of life in Zhejiang province, China (2005) [9]; and 28.7% of infants younger than 6 months of age were exclusively breastfed in twelve central and western provinces (2009) [10]. China initiated the National Basic Public Health Service Program in 2009 to improve essential public health services (including promotion of breastfeeding) in both urban and rural areas [11,12,13]. Only three studies have updated the recent national or local data on the prevalence of breastfeeding. Using the 2013 data of Chinese Nutrition and Health Surveillance, Yang et al. reported that there were 20.8%, 11.5% and 6.9% of children being breastfed at 6 months, 1 year, and 2 years of age, respectively in China [14]. Based on a local cohort study in Jiangsu Province of China (Jintan Child Health Project), Liu et al. reported 76.9% of 1656 children were exclusively breastfed for a period ≥1 month [15]. A study at Deyang region of Sichuan Province of China showed that the prevalence of exclusive breastfeeding was only 8.0% in the postnatal wards [16]. Breastfeeding prevalence rates are not available for most provinces in China currently. As revealed in many other key health indicators [17], breastfeeding prevalence may also greatly differ among provinces in China. To provide reliable input of the subnational estimation of disease burden attributed to inadequate breastfeeding, it is valuable to conduct high-quality research to report on breastfeeding prevalence at a provincial level in China. The present study aimed at reporting recent data (i.e., prevalence of children ever breastfed and exclusively breastfed for at least the first 6 months of life) based on the first Health Service Household Interview Survey of Hunan Province, China in 2013.

## 2. Methods

### 2.1. Design and Setting

Data were from the first Health Service Household Interview Survey of Hunan Province, China. This cross-sectional study was organized by the Health and Family Planning Commission of Hunan Province, formerly known as the Hunan Health Bureau, to assess the demand and utilization of health services of urban and rural residents. The survey was conducted from August to September, 2013. More details about the survey have been mentioned in recent publications [18,19]. 

### 2.2. Participants and Data Collection

Multi-stage randomized sampling was used in this survey. In the first stage, 122 counties/districts of Hunan Province were stratified into urban areas (35 districts) and rural areas (87 counties); seven counties in rural areas and seven districts in urban areas were randomly selected, respectively. Then five sub-districts in urban areas and five towns in rural areas were randomly selected from each sample district or county. Next, in each given sub-district (or town), two communities (or villages) were chosen at random. Finally, 60 households were randomly selected in each sample community (or village). All members of each selected household were invited to take part in the survey. A total of 4200 rural households and 4200 urban households were interviewed. Data was collected via face-to-face household interviews using the standardized questionnaire and procedure of the fifth National Health Service Household Interview Survey [10].

All persons who participated in data collection received two-day training and reached the criteria for conducting face-to-face household interviews. In circumstances where the interviewees were children younger than 15 years of age or in the case that adult family members were not at home at the time of the survey, parents or other adult family members answered on their behalf. The questions related to breastfeeding were answered by either parents or other adult guardians with knowledge of child-feeding.

### 2.3. Dependent Variable

The survey questionnaire has two questions regarding breastfeeding of children aged 5 years or younger: (1) Has your child ever been breastfed? (yes/no); and (2) If yes, how long was the duration of exclusive breastfeeding for him or her (excluding children who did not receive exclusive breastfeeding after birth)?

According to the definition of breastfeeding developed by the WHO [20], breastfeeding was defined as “direct from the breast or expressed breast milk” and exclusive breastfeeding was defined as “breastfeeding without any other solid or liquid (except for vitamins, mineral supplements, or medicine)”.

Two dependent variables were defined: (1) having ever been breastfed or not; (2) having been exclusively breastfed in the first six months after birth or not.

### 2.4. Independent Variables

Based on the indicators included in the survey or reported by previous reports [2,9,10,21], we included setting (urban/rural), gender, age of the child, and household income per capita as independent variables. According to a related report [22], we equally classified the households into five categories based on the household income per capita in the last year for urban areas and rural areas separately: lowest (urban, <6667 Chinese yuan (CNY); rural, <3334 CNY); lower (urban, 6667–9999 CNY; rural, 3334–4999 CNY); average (urban, 10,000–14,999 CNY; rural, 5000–7499 CNY); higher (urban, 15,000–23,999 CNY; rural, 7500–9999 CNY); and highest (urban, ≥24,000 CNY; rural, ≥10,000 CNY). 

### 2.5. Statistical Analysis

The prevalence of children having ever been breastfed and of children having been exclusively breastfed in the first six months after birth were calculated. Weights of complex sampling were considered in data analysis. We ran multivariable logistic regression to examine the associations of urban/rural setting, gender, age and household income per capita with the use of breastfeeding. Age was included as an independent variable to examine change in breastfeeding prevalence as age increased; such an analysis can be used to approximately assess whether the breastfeeding prevalence changes over time considering that usually breastfeeding ends in the first two years after the birth for almost all children. *p* < 0.05 was considered to be statistically significant. Univariable and multivariable logistic regression analyses were used to examine the associations of breastfeeding with independent variables before and after controlling for other covariates. All statistical analyses were performed using the PROC SURVEYFREQ procedure and PROC SURVEYLOGISTIC procedure of SAS 9.1 statistical software (SAS Institute Inc., Cary, NC, USA).

### 2.6. Ethics Consideration

All procedures performed in studies involving human participants were in accordance with the ethical standards of the institutional and/or national research committee and with the 1964 Declaration of Helsinki and its later amendments or comparable ethical standards. The household interview survey was approved by the Hunan Health and Family Planning Commission of Hunan Province, China. An information sheet explaining the project was given and read to the participants before obtaining their consent. The data analysis was anonymized and approved by the medical ethics committee of Central South University (reference number XYGW-2016-18).

## 3. Results

In total, 1659 of 24,282 respondents were under 5 years old and parents or other adult family members answered the survey questions for breastfeeding on their behalf.

The prevalence of children having ever received breastfeeding was 79.4% in Hunan Province, China (95% confidence interval, CI: 73.7%, 85.1%; Table 1). Differences in the prevalence of breastfeeding depending on urban/rural setting, gender and child age were insignificant, *p* > 0.05; children from households with average family income per capita were more likely to be breastfed than those from households with the lowest family income per capita, with an adjusted odds ratio of 2.28 (95% CI: 1.33, 3.91). 

Table 2 revealed that only 44.9% of children under 5 years of age were reported as having ever been exclusively breastfed in the first 6 months after birth (95% CI: 30.0%, 59.8%) (Table 2). Differences in prevalence of receiving exclusive breastfeeding were not statistically significant between urban and rural areas, nor were there statistically significant differences between boys and girls, *p* > 0.05. Children younger than 1 year and aged 3 were less often reported as being exclusively breastfed in the first six months of life than children aged 4, with adjusted odds ratios of 0.63 (95% CI: 0.42, 0.93) and 0.69 (95% CI: 0.50, 0.95), respectively. Compared to children from households with the lowest family income per capita, those from households with higher and highest family income per capita were less likely to have been exclusively breastfed in the first six months after birth, with adjusted odds ratios of 0.51 (95% CI: 0.29, 0.90) and 0.68 (95% CI: 0.46, 0.99), respectively. 

## 4. Discussion

To our knowledge, this is the first study in central south of China reporting on the latest prevalence of children having ever been breastfed and having been exclusively breastfed in the first six months after birth, using the definition of breastfeeding recommended by WHO. Our results show: (1) that 79.4% and 44.9% of children were reported as having ever been breastfed and as having been exclusively breastfed in the first six months after birth, respectively; (2) children from households with average family income per capita were more likely to be breastfed but those from households with higher and highest family income per capita were less likely to be exclusively breastfed in the first six months after birth; and (3) children younger than 1 year of age and aged 3 were reported to be less likely to have been exclusively breastfed in the first six months than children aged 4. 

The prevalence of exclusive breastfeeding in Hunan, China is higher than the global average (36%) and is approaching the goal of 50% set by the WHO [7]. Compared to previous studies in China, our study shows a high prevalence of exclusive breastfeeding in the first six months (44.9% vs. 2.6% [11], < 5% [9], 6.2 [23], 28.7% [10], and 35% [8]). The differences between this study and previous reports are likely due to many factors, such as inconsistent operational definitions for exclusive breastfeeding, geographic variations, national and local government efforts, and improved mothers’ awareness of the benefits of breastfeeding. For example, to address the challenge of the lowest prevalence of breastfeeding in the 1980s [23], China launched a nationwide campaign to promote breastfeeding in 1992 and subsequently carried out the national child development action program to promote exclusive breastfeeding [8]. From 2007, China has adopted WHO recommendations on breastfeeding [10] and has banned using breast-milk substitutes after the melamine scandal from 2008 [24]. Because of lack of data, we cannot reasonably interpret the rise of exclusive breastfeeding in the first six months after birth. Further research is warranted to explain the increased exclusive breastfeeding prevalence in Hunan Province, China. 

We found significant associations with respect to breastfeeding and exclusive breastfeeding and family income per capita. In particular, children from households with the lowest family income per capita had the lowest prevalence of breastfeeding but had the highest prevalence of exclusive breastfeeding. These findings are inconsistent with negative associations between income level and breastfeeding prevalence reported in previous studies [2,9,21]. The differences are probably due to the combined effect of individual views on the benefit of breastfeeding and family income level. Mothers from the poorest households have lower levels of health awareness for implementation of breastfeeding but have a higher chance of completing exclusive breastfeeding in the first six months than mothers from rich households because they do not face high job pressure. In China, many mothers from the poor households are jobless (particularly in rural areas), so mothers have time to implement exclusive breastfeeding for at least 6 months. In addition, poor families cannot afford expensive infant milk and thus mothers from poor families may not be willing or able to purchase infant milk and other formulas. In contrast, although many mothers from rich households have strong awareness with respect to implementation of breastfeeding for their children, they have full-time jobs and have to face high work-related pressure, and thus do not have time to commit to an exclusive breastfeeding regime in the first six months after the birth of child [9,19,25,26].

Our study did not detect significant urban-rural difference and gender difference in the prevalence of breastfeeding, which differs from previous reports in other regions of China [9,27]. The findings possibly indicate reduced urban/rural and gender differences. On the other hand, the findings may reflect the effects of differences in sampling design, operational definition, and implementation between our study and previous studies. 

In the absence of relevant information, we cannot rationally interpret differences in prevalence of exclusive breastfeeding between infants under 1 year of age and aged 4 years, and between children aged 3 and 4 years. Lower adjusted ORs for children under 1 year of age (OR = 0.63) and for children aged 3 years (OR = 0.69) seem to indicate that children who were born in 2012–2013 and those who were born in 2009–2010 were less likely to be exclusively breastfed in the first six months than children who were born in 2008–2009. These results may suggest significant changes over time in prevalence of children receiving exclusive breastfeeding at least in the first six months of life if long-time recall bias is negligible. High-quality retrospective longitudinal studies would provide solid evidence with respect to this question.

Compared to previous reports from other provinces of China, our study reported a much higher prevalence of breastfeeding and exclusive breastfeeding in Hunan province. However, we should realize that over half of all children still cannot be exclusively breastfed. This underscores that more efforts are needed to promote breastfeeding practice in Hunan Province as well as in other provinces of China. Necessary actions may include education, social support programs, and maternal and infant health protection legislation. 

This study is primarily limited by the method of data collection and survey questionnaire. Firstly, we adopted a face-to-face interview to ask parents or other family adults to recall information with respect to breastfeeding of children aged 2–5 years old; long-time recall may introduce recall bias on breastfeeding for older children as the time interval of recall increases. Secondly, this survey did not include the information relevant to breastfeeding such as reasons for not using breastfeeding, thus we cannot further explore the factors associated with not breastfeeding. Thirdly, because many parents in rural areas (particularly fathers) went to urban areas to work as migrant workers when the survey was conducted, the data on parental variables were not obtained for many fathers or mothers in rural areas, which prevented us from including more independent variables in the multivariable analysis. Another reason that we excluded other feeding variables is the lack of a matching variable that can link the information of each child to the information from the mother for the same child for the mothers who gave birth to more than two children. The exclusion of many other important factors may yield an impact on the estimation of adjusted OR. 

## 5. Conclusions

Using the data of the first Health Service Household Interview Survey of Hunan, China, we found that 79.4% and 44.9% of children were reported as having ever been breastfed and exclusively breastfed, respectively. Although the prevalence of exclusive breastfeeding in the first six months is closely approaching the goal of 50% given by WHO, more work is needed to raise the prevalence of breastfeeding, particularly exclusive breastfeeding, in Hunan, China.

## Figures and Tables

**Table 1:** Weighted prevalence of having ever been breastfed among children under 5 years of age and associations with demographic factors (Hunan Province of China, 2013).

Demographic Variable	N	Prevalence (%) (95% CI)	Unadjusted OR (95% CI)	Adjusted OR (95% CI)
Total	1659	79.4 (73.7, 85.1)		
Setting				
Urban	647	77.6 (72.9, 82.4)	1.00	1.00
Rural	1012	79.7 (73.0, 86.4)	1.13 (0.72, 1.77)	1.18 (0.76, 1.85)
Gender				
Boy	915	78.7 (73.4, 83.9)	0.91 (0.79, 1.05)	0.91 (0.78, 1.07)
Girl	744	80.3 (73.9, 86.6)	1.00	1.00
Age (year)				
<1	282	80.7 (69.6, 91.9)	0.89 (0.49, 1.62)	0.90 (0.49, 1.65)
1	364	76.3 (68.9, 83.7)	0.69 (0.42, 1.21)	0.69 (0.44, 1.09)
2	330	82.6 (72.5, 92.7)	1.01 (0.64, 1.61)	1.02 (0.64, 1.64)
3	333	75.4 (66.1, 84.7)	0.65 (0.36, 1.20)	0.70 (0.40, 1.20)
4	350	82.4 (75.9, 88.9)	1.00	1.00
Household income ^#^				
Lowest	327	70.6 (58.1, 83.1)	1.00	1.00
Lower	283	78.7 (65.7, 91.7)	1.54 (0.95, 2.49)	1.47 (0.97, 2.22)
Average	415	85.0 (78.0, 92.0)	2.36 (1.38, 4.03) *	2.28 (1.33, 3.91) *
Higher	263	83.4 (75.5, 91.4)	2.10 (0.96, 4.60)	2.11 (0.95, 4.68)
Highest	361	77.2 (72.1, 82.3)	1.41 (0.94, 2.13)	1.42 (0.92, 2.18)

CI: confidence interval; OR: odds ratio. *: *p* < 0.05. ^#^: Households were classified into five categories based on household income per capita in the last year for urban areas and rural areas separately: lowest (urban, <6667 Chinese yuan (CNY); rural, <3334 CNY); lower (urban, 6667–9999 CNY; rural, 3334–4999 CNY); average (urban, 10,000–14,999 CNY; rural, 5000–7499 CNY); higher (urban, 15,000–23,999 CNY; rural, 7500–9999 CNY); and highest (urban, ≥24,000 CNY; rural, ≥10,000 CNY). Note: Because of the presence of missing values, the sum of sample size of five household income groups was lower than the total sample size.

**Table 2:** Weighted prevalence of having ever been exclusively breastfed at least in the first 6 months of life among children under 5 years of age and associations with demographic factors (Hunan Province of China, 2013).

Demographic Variable	N	Prevalence (%) (95% CI)	Unadjusted OR (95% CI)	Adjusted OR (95% CI)
Total	1550	44.9 (30.0, 59.8)		
Setting				
Urban	595	47.2 (38.9, 55.5)	1.00	1.00
Rural	955	44.5 (35.7, 72.4)	0.89 (0.44, 1.81)	0.84 (0.42, 1.67)
Gender				
Boy	856	42.5 (25.7, 59.3)	0.81 (0.63, 1.04)	0.79 (0.58, 1.07)
Girl	694	47.8 (35.2, 60.5)	1.00	1.00
Age (year)				
<1 ^§^	173	36.3 (24.2, 48.3)	0.62 (0.40, 0.97) *	0.63 (0.42, 0.93) *
1	364	46.4 (33.5, 59.2)	0.95 (0.52, 1.74)	0.97 (0.59, 1.60)
2	330	50.9 (33.1, 68.8)	1.14 (0.82, 1.59)	1.16 (0.85, 1.58)
3	333	39.2 (23.0, 55.3)	0.71 (0.48, 1.04)	0.69 (0.50, 0.95) *
4	350	47.7 (26.3, 69.2)	1.00	1.00
Household income ^#^				
Lowest	312	52.0 (41.6, 62.3)	1.00	1.00
Lower	268	47.3 (31.6, 63.1)	0.83 (0.56, 1.23)	0.77 (0.54, 1.01)
Average	386	45.2 (17.1, 73.3)	0.76 (0.35, 1.68)	0.74 (0.35, 1.57)
Higher	246	38.0 (21.7, 54.3)	0.57 (0.33, 0.97) *	0.51 (0.29, 0.90) *
Highest	329	42.8 (29.6, 56.1)	0.69 (0.46, 1.03)	0.68 (0.46, 0.99) *

CI: confidence interval; OR: odds ratio. *: *p* < 0.05. ^§^: Children limited to ages 6–12 months. ^#^: Households were classified into five categories based on household income per capita in the last year for urban areas and rural areas separately: lowest (urban, <6667 Chinese yuan (CNY); rural, <3334 CNY); lower (urban, 6667–9999 CNY; rural, 3334–4999 CNY); average (urban, 10,000–14,999 CNY; rural, 5000–7499 CNY); higher (urban, 15,000–23,999 CNY; rural, 7500–9999 CNY); and highest (urban, ≥24,000 CNY; rural, ≥10,000 CNY). Note: Because of the presence of missing values, the sum of sample size of five household income groups was lower than the total sample size.

## Abbreviations

WHO	World Health Organization
